# Total Worker Health^®^ and Small Business Employee Perceptions of Health Climate, Safety Climate, and Well-Being during COVID-19

**DOI:** 10.3390/ijerph18189702

**Published:** 2021-09-15

**Authors:** Carol E. Brown, Lynn Dexter, Natalie V. Schwatka, Miranda Dally, Liliana Tenney, Erin Shore, Lee S. Newman

**Affiliations:** 1 Center for Health, Work & Environment, Colorado School of Public Health, University of Colorado, Anschutz Medical Campus, 13001 E. 17th Pl., 3rd Floor, Mail Stop B119, Aurora, CO 80045, USA; lynn.dexter@cuanschutz.edu (L.D.); natalie.schwatka@cuanschutz.edu (N.V.S.); miranda.dally@cuanschutz.edu (M.D.); liliana.tenney@cuanschutz.edu (L.T.); lee.newman@cuanschutz.edu (L.S.N.); 2 Department of Environmental and Occupational Health, Colorado School of Public Health, University of Colorado, Anschutz Medical Campus, 13001 E. 17th Pl., 3rd Floor, Mail Stop B119, Aurora, CO 80045, USA; 3 Department of Epidemiology, Gillings School of Public Health, University of North Carolina, 135 Dauer Drive, 2101 McGavran-Greenberg Hall, CB #7435, Chapel Hill, NC 27599-7435, USA; eshore@unc.edu; 4 Department of Epidemiology, Colorado School of Public Health, University of Colorado, Anschutz Medical Campus, 13001 E. 17th Pl., 3rd Floor, Mail Stop B119, Aurora, CO 80045, USA; 5 Division of Pulmonary Sciences and Critical Care Medicine, School of Medicine, University of Colorado, Anschutz Medical Campus, 13001 E. 17th Pl., 3rd Floor, Mail Stop B119, Aurora, CO 80045, USA

**Keywords:** Total Worker Health, leadership, health climate, safety climate, well-being, COVID-19

## Abstract

The COVID-19 pandemic created workplace challenges for employee safety and health, especially in small enterprises. We used linear mixed-effects regression to examine changes in health climate, safety climate, and worker well-being, prior to the pandemic and at two timepoints during it. We also examined whether employees at organizations that had received a TWH leadership development intervention prior to COVID-19 would better maintain pre-pandemic perceptions of climates and well-being. The final study cohort consisted of 261 employees from 31 organizations. No differences were observed in mean outcome scores between the leadership intervention groups at any of the survey timepoints. We combined intervention groups to examine the difference across timepoints. Perceptions of health and safety climates remained stable across all timepoints. However, employee well-being scores declined between the pre-pandemic period and subsequent COVID-19 timepoints. These findings suggest that while small organizations continued to be viewed as supporting employees’ health and safety over the course of the pandemic, well-being scores declined, indicating that other factors contributed to decreased well-being. The findings from this study have implications for small business leaders as they navigate the impact of the COVID-19 pandemic on the health, safety, and well-being on their organizations and employees.

## 1. Introduction

There has been a fundamental shift in working environments because of the global coronavirus pandemic (COVID-19). Research examining the impact of COVID-19 on the workplace is coming from a number of distinct fields—occupational safety and health [1,2,3,4], public health [5,6], psychology/psychiatry [7,8,9,10], business/management [11,12,13], and others [14,15]—demonstrating the multidisciplinary interest in understanding the impact of the pandemic on issues relevant to the workplace. Workplace challenges due to COVID-19 have been highlighted in the recent literature—fear of infection or disease transmission [6], job insecurity [4,5,6], struggles to maintain work–life balance [1,5,6], and mental health challenges [3,7,8,16], among others [9,12,13].

Small businesses, in particular, have faced challenges including mass layoffs, closures, and lack of cash on hand [14]. Results from an April 2020 analysis of nationally representative data showed the immediate impact of COVID-19 on small businesses, with the number of active small businesses falling by 3.3 million businesses (a 22% decrease) from February 2020 to April 2020 [12]. Losses were across almost all industries and felt most profoundly in those considered “non-essential”. Further, the losses disproportionately affected business owners who were Black, Latino, Asian, immigrant, and female [12]. Small businesses that were still operating faced additional challenges [14]. Even prior to the onset of the pandemic, smaller businesses faced higher rates of occupational injuries and illnesses [17]. They also tended to offer fewer health and safety programs, fewer benefits for workers, and had less ability to identify and mitigate workplace hazards [18].

### 1.1. Total Worker Health and Leadership

The Total Worker Health^®^ (TWH) approach, developed by the National Institute of Occupational Safety and Health (NIOSH) [19], is one framework used to address the safety, health, and well-being challenges of workers and workplaces in the time of COVID-19 [2,20]. Dennerlein and colleagues [2] outlined six characteristics that are considered best practices for the protection and promotion of worker safety, health, and well-being, the first of which is particularly relevant for the current study—leadership commitment. Leadership commitment helped foster the policies, programs, and practices that organizations put into place to address the COVID-19 pandemic [20]. Others also noted the importance of leadership to mitigate the negative effects that resulted from the uncertainty and fear surrounding COVID-19. During the pandemic, the ways in which leaders communicated changes, motivated employees, and showed empathy were associated with employee well-being [1,11]. The importance of leadership in creating a climate where employee safety and health is valued has similarly been highlighted in small businesses [21,22,23]. Our past research demonstrates a positive relationship between leadership commitment to safety and health and employees’ perceptions of safety and health climates [24] and safety and health behaviors [22].

Organizational climate is experienced by employees within an organization based on their collective perceptions of the environment, based on organizational policies, practices, and procedures [25]. Research in health climate and safety climate has consistently demonstrated the relationship between both types of climates and a number of relevant outcomes including better safety practices, increased motivation, knowledge, and fewer accidents on the safety side [26,27,28,29] and better physical health and health behaviors on the health side [30,31,32]. As outlined by Zohar, there is a demonstrated relationship between leadership and safety climate [33]. The reasons behind this relationship stem from the fact that leadership and supervisor behaviors are easy to see. When it is repeatedly observed that leaders value safety, employees report more positive perceptions of safety climate. Far less research has been conducted examining the relationship between leadership and health climate, though researchers found that leaders in organizations with high health climates were more likely to engage in health promoting leadership behaviors [34]. The TWH approach offers many ways in which workplaces impact the safety, health, and well-being of employees. A main assumption is that applying TWH in workplaces will lead to improved safety, health, and well-being outcomes. Further, if leaders are trained to apply principals of TWH leadership in the workplace, it should lead to improved safety, health, and well-being outcomes [35].

### 1.2. Study Purpose

The COVID-19 pandemic has presented researchers with an opportunity to examine what happens to health and safety climates and employee well-being during a global emergency and how TWH leadership may help leaders navigate the workplace challenges they faced starting in 2020. COVID-19 upended workplaces, creating both challenges and opportunities for occupational safety and health. During the spring of 2020, we were completing a longitudinal study examining TWH in small businesses, called the Small + Safe + Well (SSWell) Study [36]. As described below, some participating businesses were randomized to receive a TWH leadership development program intervention. Once the pandemic hit, we examined how the pandemic impacted perceptions of safety and health climates and employee well-being in the SSWell cohort of small businesses at two timepoints: during the early wave of COVID in May 2020 and in September 2020 as rates of illness in the U.S. began to rise again. In the present study, we aimed to extend our prior leadership and climate and climate research in small business [22,23,24].

First, we aimed to observe how the pandemic affected small businesses over time. A recent analysis of the state of workplace climate research has outlined the need to define the context in which climate is examined, as well as investigate multiple types of climate, and understand how climate changes over time, both naturally and through intentional attempts to modify climates [25]. While there is evidence that health and safety climates remain stable over time [33], we are unaware of evidence that examines these climates during a global emergency. We hypothesized (H1) that employees at organizations with higher safety and health climate scores and well-being scores pre-pandemic would maintain safety and health climates and well-being during the pandemic.

Second, given the evidence for the importance of leadership for workforce health, we tested the hypothesis (H2) that employees at organizations that received our TWH leadership development program prior to the pandemic would better maintain their pre-pandemic perceptions of safety climate and health climate, as well as maintain their well-being scores during the pandemic.

## 2. Materials and Methods

### 2.1. Small + Safe + Well Study (SSWell)

The SSWell parent study was a randomized, longitudinal intervention study of small businesses (<500 employees) located in the state of Colorado. The study began in April 2017, concluded in March 2020, and was housed within the Center for Health, Work and Environment (CHWE), a NIOSH Total Worker Health Center of Excellence. The SSWell study focused on the protection and promotion of worker health, safety and well-being, utilizing the TWH approach developed by NIOSH [19]. Complete details of the SSWell study have been previously described [36]. Enrolled organizations participated in Health Links™, a TWH initiative that includes assessment, certification and advising [37]. Each year of the study, a representative from each organization completed the Healthy Workplace Assessment (HWA), an organization-level survey that captures the core elements of TWH in 6 distinct realms of workplace safety and health. Following assessment, organizations were offered advising sessions to review the HWA score and to set workplace health, safety, and well-being goals for the coming year.

An additional component of the SSWell study was randomization of organizations to participate in a TWH leadership development program. The non-intervention included the HWA and advising, as described, whereas the intervention included the HWA, advising, plus participation in the TWH leadership development program. A complete description of this intervention can be found in a previous publication [38]. To briefly summarize, the TWH leadership development program included a full-day in-person training for a senior leader, who was invited to bring one additional key staff person from within the organization. Program participants completed a pre-workshop evaluation describing their TWH practices for their organization, as well as their individual personal health/wellness practices. Upon completion of the in-person training, leaders created three goals for further development over the subsequent three-month period. Leaders were also invited to participate in up to three, 30 min, individual post-training coaching sessions and to participate in an online goal setting and tracking platform.

### 2.2. Health and Safety Culture Survey

Within approximately six weeks of completion of the annual HWA, all employees at SSWell organizations were invited to complete the Employee Health and Safety Culture (HSC) Survey during each year of study enrollment. This survey included 108 items that asked employees to report on their perceptions of leadership within their organization, health and safety climates, workplace health and safety, individual well-being, as well as demographic information.

### 2.3. COVID-19 Employee Impact Surveys

We distributed a modified HSC survey to employees at active SSWell organizations at two occasions during the coronavirus pandemic. The COVID-19 Employee Impact Survey included a subset of items from the HSC survey that targeted the constructs of health climate, safety climate and employee well-being, with additional work/life questions that addressed changes that employees may have experienced because of the pandemic. Employees completed the first COVID-19 Employee Impact Survey (COVID I) in May 2020 and the second COVID-19 Employee Impact Survey (COVID II) in September 2020.

### 2.4. Health Climate, Safety Climate, and Well-Being Constructs

The health climate and safety climate constructs were developed to elicit feedback on employee perceptions of their organization’s commitment to health and safety. The health climate construct utilized items from the assessment of Zweber, Henning and Magley [30]. An example item from this four-question construct is, “My organization is committed to employee health and well-being”. Safety climate is based upon six questions from the work of Lee and colleagues [39]. An example item is, “My organization uses any available information to improve existing safety rules”. These constructs were measured using five-point Likert-type scales and were scored on a continuum of “strongly disagree” to “strongly agree” with a mean response calculated for each construct. The well-being construct included five questions from the WHO-5 Well-Being Index [40]. An example item is “I have felt cheerful and in good spirits”. This construct utilized a five-point Likert-type scale with responses ranging from “at no time” to “most of the time”. Higher mean scores on each of these constructs represent better employee perceptions of organizational health climate, safety climate, and individual well-being. All questions used in the COVID-19 Employee Impact Survey added the phrasing, “over/in the past 30 days”, in order to elicit responses specific to the pandemic timeframes that were being measured.

The HSC and COVID I and COVID II surveys were administered via email using the REDCap electronic data capture tool [41,42]. To maintain anonymity, no identifying information was collected from employees and employers were not provided employee-level response data. To incentivize participation for each survey, employees were offered the opportunity to enter random drawings for one of 15 $100 gift cards for completing surveys at each of the survey occasions. The study protocol was approved by the Colorado Multiple Institutional Review Board (COMIRB).

### 2.5. Participants

**Figure 1:**
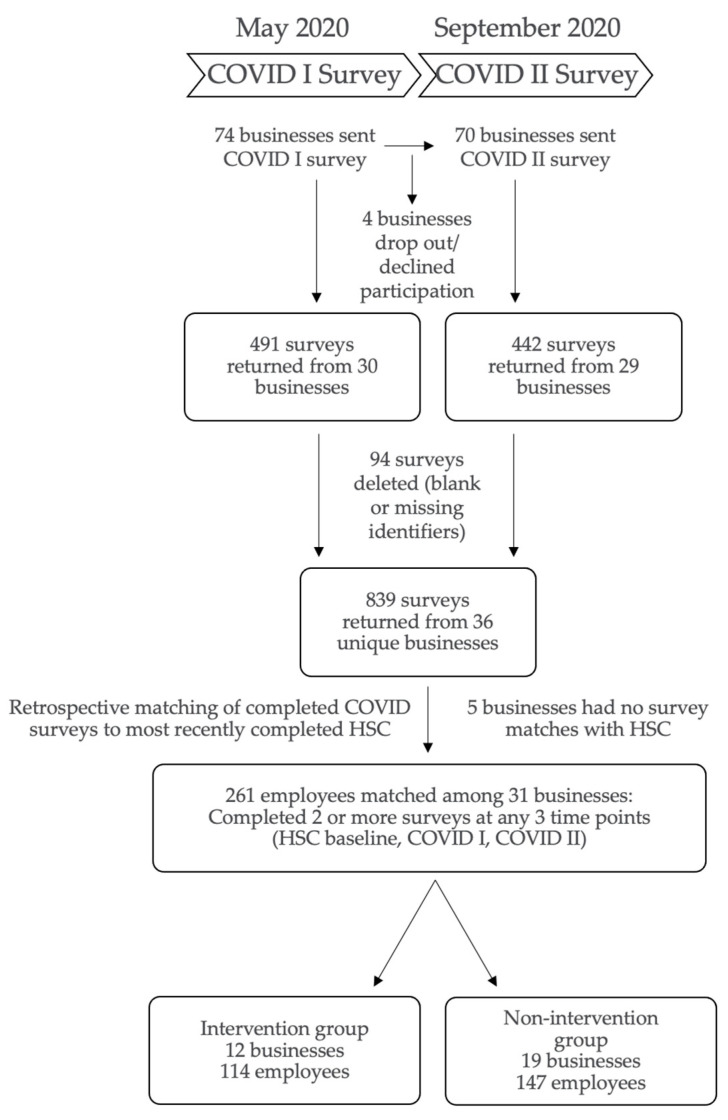
Design for the SSWell COVID-19 longitudinal study representing 31 unique businesses and 261 employees.

The study design, including number of businesses and participating employees, is displayed in Figure 1. Seventy-four of the initial 143 organizations enrolled in the SSWell study were eligible for participation in the COVID I survey in early May 2020. This included only those organizations that were considered active and had not dropped out of the study. We sent survey links to our points of contact at each site and requested that they disseminate the survey to all employees via email invitation. For the COVID I survey, we received 491 responses from employees in 30 organizations (40.5% organizational response rate). Between May and September 2020, an additional 4 organizations dropped out of the SSWell study. In early September 2020, we requested that our point of contacts at the 70 remaining active organizations send employees the COVID II survey. For the COVID II survey we received 442 responses from employees in 29 organizations (41.4% organizational response rate). We estimate that 2211 employees received the COVID I survey (22% response rate), whereas an estimated 2768 employees received the COVID II survey (16% response rate).

Across the two COVID surveys, 39 unique organizations responded. However, after deleting blank surveys, those missing unique identifiers, or responses from organizations with only a single response, the total number of responses equated to 839 employee surveys, with 36 unique organizations remaining in the study cohort. We then retrospectively matched via unique identifiers participating employees from the each COVID-19 Employee Impact Survey with their HSC responses from their most recent survey prior to the pandemic. To be included in the present study, employees must have completed any 2 of the 3 surveys from the HSC, COVID I and COVID II surveys. The final matched business cohort included 31 organizations with two or more employees completing at least two of the three surveys. We were able to match 261 employees in these organizations who had completed at least two of the three surveys. In the final cohort of 31 organizations and 261 individuals, 12 organizations with 114 matched employees previously received the leadership development program (intervention) prior to the pandemic and 19 organizations with 147 matched employees did not receive the leadership development program (non-intervention).

### 2.6. Statistical Analysis

Demographic data at the business level were collected from the most recently completed HWA, the organization-level survey competed prior to distribution of the annual HSC. Employee demographic data were collected at each of the three survey occasions and thus we present employee level demographics from first completed surveys evaluated in this study. Chi-square or Fisher exact tests were used to test for differences between categorical variables and *t*-tests with Satterthwaite corrections for unequal variances were used for continuous variables. The main outcomes of interest were changes in employees’ perceptions of health climate, safety climate and well-being across the three survey occasions. Normality of outcome variables was visually assessed.

We used linear mixed-effects regression to examine the change in the three primary outcomes over time. Random intercepts for employees nested within organization were included. Models for health climate, safety climate and well-being were assessed independently. All models were adjusted for employee age and gender. Potential confounding effects of timing of survey completion were addressed by adding a covariate for time between baseline HSC survey and first completed COVID survey. To determine if there were differences in the outcomes across survey timepoints between intervention groups, an interaction term for intervention group and time was included. Data were analyzed with SAS version 9.4 (SAS Institute Inc., Cary, NC, USA).

## 3. Results

Descriptive statistics for participating organizations and employees included in the analysis are presented in Table 1. Organizations had an average number of 84 employees (SD = 106) and 13 of 31 (41.9%) had 11–50 employees. While a range of industries were represented, the greatest proportion of organizations were from the health care and social assistance sector (*n* = 9, 29.0%) and 22 organizations (71.0%) were based in urban areas of Colorado. No demographic differences were observed for those in the intervention versus non-intervention group at the organization level.

Individual demographic data for the 261 participants indicated that employees in the intervention group were significantly younger than those in non-intervention organizations with mean ages of 34.1 years (SD = 12.7) versus 41.9 years (SD = 11.5), respectively. Most employees were white, non-Hispanic (*n* = 220, 84.3%) and female (*n* = 206, 78.9%), with no significant race/ethnicity or gender differences between intervention groups. Baseline outcome measures are presented for the 206 participants for whom we had HSC data. Results for these variables were not significantly different between the intervention groups, with mean health climate score of 4.1 (SD = 0.8), mean safety climate score of 3.9 (SD = 0.8), and mean well-being score equal to 3.6 (SD = 0.7).

Mean time from baseline HSC to first completed COVID survey differed between the groups, with the intervention group averaging 281 days (SD = 98) and 370 days (SD = 101) in the non-intervention group (*p* < 0.0001). Mean number of days from the TWH leadership development program to first completed COVID survey was 406 days (SD = 143) for those organizations in the intervention group.

### Mixed-Effects Linear Regression Analysis

**Figure 2:**
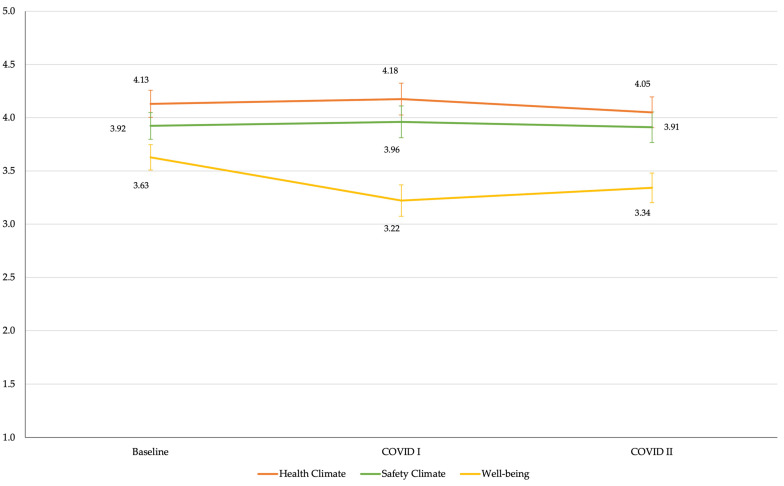
Least squares mean estimation of health climate, safety climate, and employee well-being at each timepoint.

Table 2 presents least squares means for health climate, safety climate and well-being by intervention group at each survey timepoint. No differences were observed in mean outcome scores between the intervention and non-intervention groups at any of the survey timepoints shown. Figure 2 presents the least squares mean estimation and 95% CI for health climate, safety climate and well-being at each survey timepoint for intervention groups combined.

Table 3 presents the change in outcomes across the three timepoints, for the two intervention groups combined. Health climate and safety climate remained stable across the three timepoints. However, well-being scores declined between the baseline HSC survey and the COVID I and the COVID II surveys. From the HSC survey to the first COVID survey, well-being scores (out of 5 points) declined by −0.41 points (*p* < 0.0001, 95% CI = −0.558, −0.253). From the baseline HSC survey to the COVID II survey, mean well-being scores decreased by −0.29 points (*p* < 0.001, 95% CI = −0.435, −0.137).

To test for introduction of potential bias between included and excluded organizations, we assessed organization level differences in demographics of employee participants from the 31 participating versus 43 non-participating organizations. There were no significant differences in mean number of employees, business size, industry, or region among the 74 eligible SSWell organizations comparing the 31 organizations that were included in this analysis with the 43 excluded organizations (Supplementary Table S1).

We included in the analyses the 261 employees at the 31 organizations who had completed at least two of the three surveys. This resulted in missing data across surveys (Supplementary Table S2). To evaluate the effect of missing data, we compared our outcome variables for the respondents completing all three surveys (*n* = 54) to the respondents completing any of two of the surveys (*n* = 207). No significant differences were observed in mean health climate, safety climate or well-being for those with only two completed surveys compared to employees who completed all three surveys (Supplementary Table S3).

## 4. Discussion

In the first year of COVID-19, small businesses demonstrated the ability to sustain employee perceptions of health and safety climates at pre-COVID-19 levels. Health climate and safety climate scores remained stable across both groups and all three timepoints. Nonetheless, employees reported worsening of well-being, suggesting that factors other than health and safety climate likely contributed to their perceptions of well-being during the pandemic. Our hypothesis that TWH leadership training would enable businesses to better maintain their pre-pandemic perceptions of safety climate and health climate, and for their employees to maintain their well-being scores during the pandemic was not supported by the results. Self-reported employee well-being scores declined in both the TWH leadership program intervention group and in the non-intervention group between baseline and COVID I and COVID II timepoints.

### 4.1. Theoretical Contributions

The stability in health climate and safety climate over time is consistent with previous literature in the safety climate field. As safety climate builds through social interactions over time, it stands that it would be somewhat stable and not easily changed [33,43]. There is an identified need to more closely examine the stability of health and safety climates over time and in relation to external factors [25] and the current study adds to this evidence base.

The decline in worker well-being scores is consistent with other recent findings. One study, conducted at an academic institution, found 58.3% of respondents reported worsened well-being related to COVID-19 work and non-work changes [3]. Multiple other studies conducted over the course of the pandemic have reported worsened mental health outcomes because of COVID-19. For example, a narrative review that included 35 studies examining psychological problems related to the workplace during COVID-19 reported high rates of depression, anxiety, and stress among workers from a wide range of occupations and from multiple countries [16].

The present study offers a unique look into climate perceptions during COVID-19. Historically, climate researchers have been focused on survey development [44] and relating climate perceptions to important antecedents and outcomes [45]. If changes in climate perceptions have been assessed, it has been because researchers were testing interventions. This may be due, in part, to differences in research traditions where culture researchers tend to examine the evolution of systems over time whereas climate researchers tend to focus on the impact of social systems [46]. In the present study, we begin to close this gap. Our study demonstrates that over a period of about one year, safety and health climates are relatively stable. To our surprise, their stability over time occurred even in the face of a global pandemic. This effect may be due, in part, to a selection bias whereby our sample included small businesses still in operation and willing to continue in a research study during a pandemic. Such businesses may be in a better position to maintain their health and safety practices in general.

Prior research suggests that climate perceptions change when leadership and communication practices are instituted [35]. As such, we had hypothesized that a TWH leadership development program would help small businesses maintain their climates and for employees to maintain their well-being. Health and safety climates during COVID-19 continue to be important, as organizations and leaders aim to promote the new health and safety procedures that have resulted from COVID-19 [10]. The results from our initial, cross-sectional study demonstrated that employees in small businesses reported better well-being during the initial wave of COVID-19 when they worked for an organization that they perceived had strong health and safety climates [47]. In fact, no other work or life factors, such as changes to childcare or limiting social contacts, were significant after accounting for perceptions of health and safety climates. We can only speculate as to why TWH leadership training failed to impact well-being. For example, the “dose” of leadership training may have been insufficient [35,48], training transfer may not have occurred [35], the size of our study may have been too small to detect a benefit, the multiple ways in which the pandemic affected well-being may have neutralized any leadership effect, or TWH leadership training does not contribute to worker well-being.

### 4.2. Practical Applications

The TWH leadership development program that served as the intervention for this study was not designed to address the health and safety needs of workers during a global pandemic. However, many of the concepts of the training were relevant to supporting the health and safety climates of organizations that could serve as a foundation in the event of an emergency. There are lessons we can take from the current study and other recent findings to adapt the TWH leadership development program. The literature from the fields of management and psychology focuses on communication by leaders as a key area that will help support employees during COVID-19 [1,11]. Specifically, regular communication, transparency, leading by example, and empathy are identified as specific leader behaviors that are especially important during this time. Other researchers have highlighted the key role that supervisory support plays [1,3]. Since leadership is one key area that is related to improved health and safety climates [33,34], we need to continue to develop the TWH leadership skills for small business leaders. One practical challenge is that the “dose” of leadership training that may be needed to change behavior may not match the time and effort that small business leaders are willing to commit to TWH.

Taken together, the findings from this study have real-world implications for small business leaders who continue to navigate the impact of the COVID-19 pandemic on the health, safety, and well-being on their organizations and their employees. One such implication is the need to focus on employee well-being more holistically. It is possible that even when workplace health and safety climates are generally positive, employee well-being may worsen due to other work and non-work factors. Already, research has demonstrated that caregivers of young children are experiencing high levels of pandemic stress which is related to worse mental health symptoms [49]. Our study cannot point to the specific factors that may have influenced the observed decline in well-being. However, our study does suggest that organizations and leaders have an opportunity to learn about and address employee needs to better understand the decline in well-being.

### 4.3. Future of Work

COVID-19 brought to the forefront several priority topics previously highlighted by NIOSH in putting forth a vision of the future of work and improving workforce safety, health, and well-being [50]. The COVID-19 pandemic led to an increase in changes to organizational design, including increased numbers of remote workers; need for work–life fit; changes to the physical work environment; and a re-examination of benefits, especially leave and bereavement benefits. As noted in their foundational paper, Tamers and colleagues state that as workplaces, work, and workforces change, organizational leadership has an important role to play in protecting and promoting the safety, health, and well-being of workers [50]. While the results from this study did not find a significant relationship between prior participation in TWH leadership development and employee perceptions of health climate, safety climate, and well-being, we did find that worker well-being decreased over time through the pandemic and that both work and non-work factors are likely contributors to this decline. Further, the demographics of the workforce are continuing to change. Workers from racial and ethnic minorities already faced increased work-related health disparities, vulnerabilities, and stress [51]. COVID-19 has disproportionately impacted workers of color [12,52]. It was beyond the scope of this study to examine differences in perceived climates and well-being, based on respondents’ race and ethnicity. However, this will continue to be an important focus for future studies.

### 4.4. Future Research Directions

The COVID-19 pandemic response has accelerated changes in the nature of work and is likely to continue to impact workforce health, safety and well-being for the foreseeable future. Multiple researchers have highlighted the role occupational safety and health professionals will continue to have as we emerge from the pandemic and work to create healthy and safe workplaces [10,13,53]. There will be a need to study the impact of COVID-19 on workplace climate and its relationship to worker well-being. Researchers should continue to study how the different phases of the pandemic impact employee health, safety, and well-being and the impact of the new policies, programs, and practices that are put into place to address workplace and employee needs [3,5]. Chang and colleagues [10] outline eight areas for designing workplace interventions that leaders can implement to help employees cope with COVID-19, including physical health and safety; work–life balance; job security; well-being; training and skill development; virtual work and alternate work arrangements; recognition; and involvement. These areas overlap with many of the relevant issues that the TWH approach outlined to advance worker well-being [54] as well as priorities highlighted in the NIOSH future of work initiative [50].

### 4.5. Strengths and Limitations

Due to the ongoing SSWell study, we were able to quickly develop and deploy the COVID-19 Employee Impact Survey that built upon our previous study materials and distribute it to our participating study organizations, increasing the speed by which we could collect data. The longitudinal nature of this study allowed us to assess health and safety climates and well-being at three timepoints, before and during the COVID-19 pandemic. The employees in the study represented organizations of various small business sizes, industries, and regions of the state, increasing the generalizability of our findings. Limitations must also be noted and include the nature of the organizations that were still operating and the employees who were able to complete the surveys. Organizations that may have shut down and employees who were furloughed or no longer working would not have been represented in this study. Based on the nature of the organizations enrolled in the study and the data collection methods, it is likely that employees surveyed were more likely to represent white collar workers. A sample that specifically examined differences by industry and/or included more frontline workers may have led to different results. The data collected were self-reported and though our questions asked participants to limit their responses to the previous 30 days, the heightened stress and time of great change may have affected participant recall.

## 5. Conclusions

Employee perceptions of health and safety climates remained relatively stable from the pre-COVID-19 baseline period to May and September of 2020. This, in part, demonstrates that organizations, even in the face of upheaval caused by the COVID-19 pandemic, maintained stable environments for their employees. In other words, the pandemic did not appear to change employees’ perceptions of health climate or safety climate. However, the lower employee well-being scores indicate that work and other non-work factors are likely contributing to diminished employee well-being. As of September 2020, COVID-19 was still having a major impact on small businesses. Organizations have a real opportunity to develop and implement innovative supports for employees in the changing nature of the workplace.

## Figures and Tables

**Table 1:** Business characteristics and employee demographics by intervention group.

Business Characteristics	Combined (*n* = 31)	Intervention (*n* = 12)	Non-Intervention (*n* = 19)	*p*-Value
	*N* (%)/Mean (SD)	*N* (%)/Mean (SD)	*N* (%)/Mean (SD)	
Number of Employees, Mean (SD)	84 (106)	87 (87)	83 (119)	0.92
Business Size				0.17
Micro (2–10 employees)	6 (19.4%)	0 (0.0%)	6 (31.6%)	
Small (11–50 employees)	13 (41.9%)	7 (58.3%)	6 (31.6%)	
Medium (51–200 employees)	6 (19.4%)	3 (25.0%)	3 (15.8%)	
Large (>200 employees)	6 (19.4%)	2 (16.7%)	4 (21.1%)	
Industry				0.54
Health Care and Social Assistance	9 (29.0%)	6 (50.0%)	3 (15.8%)	
Non-Profit	5 (16.1%)	1 (8.3%)	4 (21.1%)	
Educational Services	4 (12.9%)	1 (8.3%)	3 (15.8%)	
Public Administration	4 (12.9%)	1 (8.3%)	3 (15.8%)	
Arts, Entertainment and Recreation	2 (6.5%)	1 (8.3%)	1 (5.3%)	
Construction	2 (6.5%)	1 (8.3%)	1 (5.3%)	
Real Estate and Rental and Leasing	2 (6.5%)	0 (0.0%)	2 (10.5%)	
Accommodation and Food Service	1 (3.2%)	1 (8.3%)	0 (0.0%)	
Services	1 (3.2%)	0 (0.0%)	1 (5.3%)	
Other	1 (3.2%)	0 (0.0%)	1 (5.3%)	
Region				0.42
Urban	22 (71.0%)	10 (83.3%)	12 (63.2%)	
Rural	9 (29.0%)	2 (16.7%)	7 (36.8%)	
**Employee Demographics**	**Combined** **(*n* = 261)**	**Intervention** **(*n* = 114)**	**Non-Intervention** **(*n* = 147)**	***p*-Value**
Age (years)	38.5 (12.6)	34.1 (12.7)	41.9 (11.5)	<0.0001
Race/Ethnicity				0.32
White, non-Hispanic	220 (84.3%)	93 (81.6%)	127 (86.4%)	
Black or African American	5 (1.9%)	4 (3.5%)	1 (0.7%)	
Hispanic, Latino, Spanish Origin	26 (10.0%)	11 (9.7%)	15 (10.2%)	
Asian	2 (0.8%)	1 (0.9%)	1 (0.7%)	
Other/Multiracial	5 (1.9%)	4 (3.5%)	1 (0.7%)	
Did not provide	3 (1.2%)	1 (0.7%)	2 (1.4%)	
Gender				0.39
Male	54 (20.7%)	21 (18.4%)	33 (22.5%)	
Female	206 (78.9%)	92 (80.7%)	114 (77.6%)	
Other	1 (0.4%)	1 (0.9%)	0 (0.0%)	
**Baseline HSC Study Measures, *N* = 206**	**Mean (SD)**	**Intervention** **(*n* = 88)**	**Non-Intervention** **(*n* = 118)**	***p*-Value**
Health climate	4.1 (0.8)	4.1 (0.8)	4.0 (0.8)	0.37
Safety climate (*n* = 202)	3.9 (0.8)	3.8 (0.8)	3.9 (0.8)	0.55
Well-being (*n* = 199)	3.6 (0.7)	3.7 (0.7)	3.5 (0.7)	0.09
Time from baseline HSC to first completed COVID survey, mean number of days (SD)	332 (109)	281 (98)	370 (101)	<0.0001
Time from TWH intervention to first completed COVID survey, mean number of days (SD)	NA	406 (143)	NA	NA

**Table 2:** Least squares mean estimation of health climate, safety climate, and well-being at each survey occasion stratified by intervention group.

		Intervention	Non-Intervention		
Variable	Time	Mean (95% CI)	Mean (95% CI)	Difference	*p*-Value
Health Climate	Baseline	4.05 (3.734, 4.358)	4.18 (3.949, 4.416)	−0.14	0.58
	COVID I	4.07 (3.748, 4.393)	4.24 (3.980, 4.500)	−0.17	0.50
	COVID II	3.93 (3.590, 4.261)	4.13 (3.884, 4.378)	−0.21	0.42
Safety Climate	Baseline	3.68 (3.371, 3.981)	4.09 (3.863, 4.322)	−0.42	0.08
	COVID I	3.86 (3.546, 4.180)	3.99 (3.728, 4.243)	−0.12	0.62
	COVID II	3.76 (3.430, 4.090)	4.02 (3.780, 4.267)	−0.26	0.30
Well-Being	Baseline	3.70 (3.422, 3.981)	3.57 (3.355, 3.783)	0.13	0.54
	COVID I	3.19 (2.902, 3.488)	3.26 (3.012, 3.504)	−0.06	0.78
	COVID II	3.32 (3.014, 3.632)	3.35 (3.115, 3.574)	−0.02	0.92

**Table 3:** Mixed-effects linear regression analyses showing change in health climate, safety climate and well-being scores at three survey occasions, *N* = 261.

	Baseline HSC to COVID I		COVID I to COVID II		Baseline to COVID II	
Variable	Estimate (95% CI)	*p*-Value	Estimate (95% CI)	*p*-Value	Estimate (95% CI)	*p*-Value
Health Climate	0.05 (−0.091, 0.181)	0.52	−0.12 (−0.285, 0.035)	0.13	−0.08 (−0.213, 0.053)	0.24
Safety Climate	0.04 (−0.102, 0.179)	0.59	−0.05 (−0.216, 0.114)	0.54	−0.01 (−0.150, 0.125)	0.86
Well-Being	−0.41 (−0.558, −0.253)	<0.0001 *	0.12 (−0.056, 0.295)	0.18	−0.29 (−0.435, −0.137)	0.0002 **

Note. Models controlled for age, gender, and time from baseline HSC to first completed COVID-19 Employee Impact Survey. * Significant at *p* < 0.0001. ** Significant at *p* < 0.001.

## Data Availability

The de-identified data presented in this study are available upon request from the corresponding author.

## Supplementary Materials

The following are available online at https://www.mdpi.com/article/10.3390/ijerph18189702/s1. Table S1. Demographic data comparing organizations meeting study inclusion criteria compared to excluded organizations. Table S2. Distribution of completed surveys among 261 unique respondents. Table S3. Comparison of mean health climate, safety climate and well-being scores for employees completing two surveys versus employees completing three surveys.
