# Supplementary material for: Total Worker Health® and Small Business Employee Perceptions of Health Climate, Safety Climate, and Well-Being during COVID-19
Source: Int J Environ Res Public Health. 2021 Sep 15;18(18):9702. doi: 10.3390/ijerph18189702 (PMC8469982; doi:10.3390/ijerph18189702)
Supplement: Supplementary file 1 [file ijerph-18-09702-s001.zip › ijerph-1352298-supplementary.pdf]

Table S1. Demographic data comparing organizations meeting study inclusion criteria compared to excluded organizations.

Business Characteristics, N=74.	N (%) or Mean (SD)	Excluded Organizations (n=43)	Included Organizations (n=31)	p-value
Number of Employees, mean (SD)	83 (87)	87 (77)	77 (100)	0.63
Business size				0.50
Micro (2-10 employees)	11 (14.9%)	5 (11.6%)	6 (19.4%)	
Small (11-50 employees)	31 (41.9%)	17 (39.5%)	14 (45.2%)	
Medium (51-200 employees)	21 (28.4%)	15 (34.9%)	6 (19.4%)	
Large (200+ employees)	11 (14.9%)	6 (14.0%)	5 (16.1%)	
Industry				0.16
Accommodation & Food Service	2 (2.7%)	1 (2.3%)	1 (3.2%)	
Administration, Support & Waste Management	1 (1.4%)	1 (2.3%)	0 (0.0%)	
Agriculture, Forestry, Fishing, Hunting	2 (2.7%)	2 (4.7%)	0 (0.0%)	
Arts, Entertainment & Recreation	3 (4.1%)	1 (2.3%)	2 (6.5%)	
Construction	6 (8.1%)	4 (9.3%)	2 (6.5%)	
Educational Services	7 (9.5%)	3 (7.0%)	4 (12.9%)	
Healthcare and social assistance	15 (20.3%)	6(14.0%)	9 (29.0%)	
Information	1 (1.4%)	1 (2.3%)	0 (0.0%)	
Manufacturing	5 (6.8%)	5 (11.6%)	0 (0.0%)	
Non-profit	7 (9.5%)	2 (4.7%)	5 (16.1%)	
Public Administration	8 (10.8%)	4 (9.3%)	4 (12.9%)	
Real Estate & Rental & Leasing	3 (4.1%)	1 (2.3%)	2 (6.5%)	
Retail/Wholesale Trade	1 (1.4%)	1 (2.3%)	0 (0.0%)	
Services	9 (12.2%)	8 (18.6%)	1 (3.2%)	
Transportation, Warehousing & Utilities	1 (1.4%)	1 (2.3%)	0 (0.0%)	
Other	3 (4.1%)	2 (4.7%)	1 (3.2%)	
Region				0.46
Urban	49 (66.2%)	27 (62.8%)	22 (71.0%)	
Rural	25 (33.8%)	16 (37.2%)	9 (29.0%)	

Table S2. Distribution of completed surveys among 261 unique respondents.

Baseline Health Culture Survey	COVID I Survey	COVID II Survey	Matched n=261	Total Surveys n= 576
	X	X	55	110
X		X	78	156
X	X		74	148
X	X	X	54	162

Table S3. Comparison of mean health climate, safety climate and well-being scores for employees completing 2 surveys versus employees completing 3 surveys.

	Baseline Health and Safety Culture Survey			COVID I Survey			COVID II Survey		
	2 Surveys Completed	3 Surveys Completed	p-value	2 Surveys Completed	3 Surveys Completed	p-value	2 Surveys Completed	3 Surveys Completed	p-value
Health climate	4.0 (0.81)	4.2 (0.76)	0.37	4.1 (0.85)	4.1 (0.85)	0.94	4.1 (0.78)	4.0 (0.89)	0.61
Safety climate	3.8 (0.79)	3.9 (0.71)	0.45	3.9 (0.83)	3.9 (0.80)	0.72	3.9 (0.84)	3.9 (0.85)	0.71
Well-being	3.5 (0.69)	3.6 (0.74)	0.31	3.1 (0.84)	3.1 (0.89)	0.92	3.3 (0.87)	3.3 (0.74)	0.57
